# Kidney tea [*Orthosiphon aristatus* (Blume) Miq.] improves diabetic nephropathy via regulating gut microbiota and ferroptosis

**DOI:** 10.3389/fphar.2024.1392123

**Published:** 2024-06-19

**Authors:** Zheng Zhou, Hongjuan Niu, Meng Bian, Chunsheng Zhu

**Affiliations:** ^1^ Department of Chinese Medicine, The First Affiliated Hospital of Zhengzhou University, Zhengzhou, China; ^2^ Center for Reproductive Medicine, Department of Obstetrics and Gynecology, Peking University Third Hospital, Beijing, China

**Keywords:** *Orthosiphon aristatus* (Blume) Miq., diabetic nephropathy, gut microbiota, serum metabolites, ferroptosis

## Abstract

**Introduction:**

Diabetic nephropathy (DN) is the leading cause of end-stage renal disease. Due to its complex pathogenesis, new therapeutic agents are urgently needed. *Orthosiphon aristatus* (Blume) Miq., commonly known as kidney tea, is widely used in DN treatment in China. However, the mechanisms have not been fully elucidated.

**Methods:**

We used db/db mice as the DN model and evaluated the efficacy of kidney tea in DN treatment by measuring fasting blood glucose (FBG), serum inflammatory cytokines, renal injury indicators and histopathological changes. Furthermore, 16S rDNA gene sequencing, untargeted serum metabolomics, electron microscope, ELISA, qRT-PCR, and Western blotting were performed to explore the mechanisms by which kidney tea exerted therapeutic effects.

**Results:**

Twelve polyphenols were identified from kidney tea, and its extract ameliorated FBG, inflammation and renal injury in DN mice. Moreover, kidney tea reshaped the gut microbiota, reduced the abundance of *Muribaculaceae*, *Lachnoclostridium*, *Prevotellaceae_UCG-001*, *Corynebacterium* and *Akkermansia*, and enriched the abundance of *Alloprevotella*, *Blautia* and *Lachnospiraceae_NK4A136_group*. Kidney tea altered the levels of serum metabolites in pathways such as ferroptosis, arginine biosynthesis and mTOR signaling pathway. Importantly, kidney tea improved mitochondrial damage, increased SOD activity, and decreased the levels of MDA and 4-HNE in the renal tissues of DN mice. Meanwhile, this functional tea upregulated GPX4 and FTH1 expression and downregulated ACSL4 and NCOA4 expression, indicating that it could inhibit ferroptosis in the kidneys.

**Conclusion:**

Our findings imply that kidney tea can attenuate DN development by modulating gut microbiota and ferroptosis, which presents a novel scientific rationale for the clinical application of kidney tea.

## Graphical Abstract

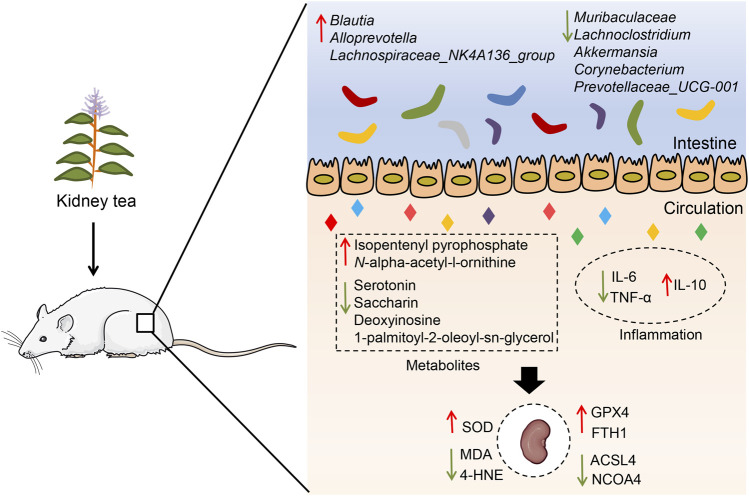

## 1 Introduction

Diabetes and its various complications seriously threaten the health of patients and impose a huge medical and economic burden on society. According to statistics, there were approximately 537 million adults with diabetes globally in 2021, and this number will rise to 783 million by 2045 (Sun et al., 2022). Diabetic nephropathy (DN) is one of the major microvascular complications of diabetes, accounting for 40% of diabetic patients (Smyth et al., 2022). It is mainly characterized by hypertension, progressive proteinuria, and decreased renal function, ultimately developing into end-stage renal disease. Increasing evidence suggests that multiple pathological mechanisms, such as oxidative stress (Liu et al., 2023), chronic inflammation (Qiu et al., 2023), mitochondrial dysfunction (Zhou et al., 2023) and excessive ferroptosis (Li et al., 2023), play crucial roles in the occurrence and progression of DN. Currently, the therapeutic strategies that limit the development of DN include anti-hyperglycemic sodium-glucose cotransporter 2 (SGLT2) inhibitors, as well as anti-hypertensive angiotensin receptor blockers (ARBs) and angiotensin-converting enzyme inhibitors (ACEIs) (Zhang et al., 2022a; Barrera-Chimal et al., 2022; Vergara et al., 2023). Due to the complex pathological mechanisms, it is urgent to develop effective agents for DN management.

*Orthosiphon aristatus* (Blume) Miq., also known as kidney tea, is a perennial herb belonging to the family Lamiaceae (Zhu et al., 2023). Kidney tea is recorded in the traditional Dai medical works “Bei Ye Jing” and “Dang Haya,” and has been used for more than 2,000 years. It is traditionally consumed as a functional tea in southwestern China and Southeast Asia, owing to its distinctive aromatic odour (Ashraf et al., 2018). On the other hand, this time-honoured medicine is widely used to treat kidney diseases, gout and diabetes (Hsu et al., 2010; Ching et al., 2013; Chen et al., 2020). In a previous study, we identified five compounds from kidney tea and confirmed that rosmarinic acid might play a hypoglycemic effect by regulating the activity of α-glucosidase (Zhu et al., 2021). Another research found that aqueous and methanolic extracts of kidney tea could reduce blood glucose levels in diabetic mice via increasing glucose transporter 4 (GLUT4) translocation to the plasma membrane (Bassalat et al., 2023). Furthermore, the phenolic acids might be the active ingredients of kidney tea that exerted antidiabetic effects, and the mechanisms were related to antioxidant activity, inhibition of α-glucosidase and α-amylase, and phosphatidylinositol 3-kinase (PI3K)/protein kinase B (AKT) pathway-mediated glucose uptake (Wang et al., 2023). However, the mechanisms by which kidney tea improves DN have not been elucidated.

Gut microbiota is the microorganisms in the human gastrointestinal tract, which is vital for maintaining the health of the host. A large number of studies have found that the gut microbiota is involved in the pathogenesis of DN (Zhang et al., 2022b; Cai et al., 2022; Zhao et al., 2023). DN patients exhibited dysbiosis in the gut microbiota, manifested by increased levels of *Lactobacillus*, *Megaphaera* and *Sutterella*, as well as decreased levels of *Lachnochlostridium* and *Roseburia* (Du et al., 2021). Recent research has demonstrated that gut microbiota-derived outer membrane vesicles can promote DN progression by activating tubulointerstitial inflammation (Chen et al., 2023). Notably, the diversity of the gut viruses was significantly reduced in DN patients (Fan et al., 2023). In addition, non-targeted metabolomics revealed that plasma kynurenine, gluconolactone, CE-C0218 and CE-A0242, as well as urinary threonic acid, sphingomyelin, 1-methylpyridin-1-ium and 1-palmitoyl-glycero-3-phosphocholine could be potential biomarkers for DN (Hirakawa et al., 2022). These findings imply that the changes in the composition of gut microbiota and metabolites are involved in the pathogenesis of DN, and that interventions targeting them may provide new therapeutic strategies for DN. Here, we hypothesized that kidney tea had the potential to regulate gut microbiota and host metabolic function, thereby exerting a therapeutic effect on DN. Therefore, we investigated the protective effects of kidney tea on DN mice and elucidated the possible mechanisms by combining gut microbiota and serum metabolomics analysis.

## 2 Materials and methods

### 2.1 Sample preparation

Kidney tea was collected from Yunnan Province, China, and then identified by Prof. Binghong Fei. Kidney tea (600 g) was soaked in 6,000 mL of water for 1 h, followed by decoction for 30 min and filtration. This process was repeated twice, and the combined extract was concentrated to 1 g/mL using a rotary evaporator and then stored in a refrigerator at −20°C for subsequent experiments.

### 2.2 Qualitative analysis of kidney tea

The phytochemical profiles of kidney tea were carried out with ultrahigh-performance liquid chromatography (UHPLC)-mass spectrometry (MS). Thermo UHPLC vanquish (Thermo Fisher Scientific) was used for chromatographic separation with an ACQUITY UPLC HSS T3 column (100 mm × 2.1 mm, 1.8 μm) at 35°C. Water containing 0.1% formic acid and acetonitrile solution containing 0.1% formic acid were served as mobile phases A and B, respectively. The gradient program of mobile phase B was as follows: 0–17.0 min, 5%–98%; 17.0–17.2 min, 98%–5%; 17.2–20.0 min, 5%. The injection volume was 2 μL with a flow rate of 0.3 mL/min.

Thermo Q-Exactive HFX (Thermo Fisher Scientific) was used for MS analysis, and the electrospray ionization (ESI) source was used for detection in positive and negative ion modes. The parameters of ESI source were set as follows: the spray voltage was 3.8 kV (+) and 3.0 kV (−), the source temperature was 320°C, and the range of MS scan was 90–1,300 m/z.

### 2.3 Animals and drug intervention

The animal experiment was approved by the Life Science Ethics Committee of the First Affiliated Hospital of Zhengzhou University (No. 2024-KY-0015-002). 30 male C57BLKS/J db/db and 6 male C57BLKS/J db/m mice (7-week-old) were obtained from Nanjing Junke Bioengineering Co., Ltd. (Nanjing, China). db/db mouse exhibits leptin receptor deficiency and often serves as a common animal model for spontaneous type 2 diabetes (Suriano et al., 2021). All mice were maintained in an SPF-grade environment with a temperature of 22°C ± 2°C, a relative humidity of 50% ± 5%, and a 12 h light/dark cycle. All animals had free access to standard feed and water with 1 week of acclimatization. Subsequently, 30 db/db mice were randomly allocated into five groups (*n* = 6), namely, the model group (Mod), the kidney tea low-dose group (KTL), the kidney tea medium-dose group (KTM), the kidney tea high-dose group (KTH), and the dapagliflozin group (DAPA). Moreover, 6 db/m mice were used as the control group (Con). Of these, the KTL, KTM and KTH groups were given 2, 4 and 8 g·kg^−1^·d^−1^ kidney tea extract, respectively. The DAPA group was given 1.3 mg·kg^−1^·d^−1^ dapagliflozin. After 1 week of adaptive feeding, the mice in each intervention group received the corresponding drug, and the mice in the Con and Mod groups received distilled water. All mice were gavaged with a volume of 10 mL·kg^−1^ and administered once daily for 8 weeks.

### 2.4 Sample collection

The blood from the tail vein was collected after an 8-hour fast at 0, 2, 4, 6, and 8 weeks, and the fasting blood glucose (FBG) levels were measured by a glucometer (Bayer Healthcare LLC, NY, United States). The urine was collected at week 8 by metabolic cages. Ultimately, the mice were anaesthetized with pentobarbital sodium, the eyeballs were removed for blood collection, and serum was obtained by centrifugation. After the mice were executed, the kidneys and contents from the ileocecal junction were collected.

### 2.5 Measurement of inflammatory cytokines and renal injury indicators

Serum tumor necrosis factor-α (TNF-α), interleukin-6 (IL-6), and IL-10 levels were detected with ELISA kits purchased from Jiangsu Enzyme Labeling Biotechnology Co., Ltd. (Yancheng, China). Blood urea nitrogen (BUN) and serum creatinine (Scr) levels were detected by an automatic biochemical analyzer (Rayto Life and Analytical Sciences Co., Ltd., Shenzhen, China). Urine *N*-acetyl-β-D-glucosaminidase (NAG), albumin, and neutrophil gelatinase-associated lipocalin (NGAL) levels were detected by assay kits purchased from Nanjing Jiancheng (Nanjing, China) to monitor renal injury.

### 2.6 Histopathologic examination

The kidney tissues of appropriate size were fixed in 4% paraformaldehyde, and the fixed tissues were embedded in paraffin. The samples were sectioned and stained with hematoxylin and eosin (H&E), periodic acid-Schiff (PAS) and Masson, and subsequently observed under light microscope for renal histopathological changes.

### 2.7 16S rDNA gene sequencing

Total microbial DNA was extracted from the contents of the ileocecal junction of mice by a Magnetic Soil And Stool DNA Kit (Tiangen Biochemical Technology Co., Ltd., Beijing, China). Amplification of 16S rDNA gene (V3-V4 regions) was performed with the specific primers 341F (5′-CCTACGGGNGGCWGCAG-3′) and 806R (5′-GGACTACHVGGGTATCTAAT-3′) with barcodes. After electrophoresis and purification, the purified amplicons were sequenced by Illumina NovaSeq 6000 platform (Illumina, San Diego, CA, United States).

The FASTQ format data were processed through QIIME 2 pipeline. Taxonomic assignment of amplicon sequence variants (ASVs) was performed based on the SILVA v138 16S database. The composition of species in the samples was analyzed based on the results of species annotation. Principal coordinates analysis (PCoA) was used to assess the beta diversity, and linear discriminant analysis effect size (LEfSe) analysis was performed to further screen significantly different species within different groups.

### 2.8 Untargeted metabolomics analysis

The serum processing method and instrument details in the untargeted metabolomics analysis are reported in our previous literature (Zhu et al., 2023). The mobile phase was a mixture of solvent A (acetonitrile) and solvent B (water containing 25 mM ammonium acetate and 25 mM ammonium hydroxide) at a flow rate of 0.5 mL/min. The gradient program was as follows: 0–0.5 min, 5% B; 0.5–7.0 min, 5%–35% B; 7.0–8.0 min, 35%–60% B; 8.0–9.0 min, 60% B; 9.0–9.1 min, 60%–5% B; 9.1–12.0 min, 5% B.

Orthogonal partial least squares discriminant analysis (OPLS-DA) and principal component analysis (PCA) were performed to evaluate the overall distribution trend among groups. Variable importance in the projection (VIP) value > 1 and *p* < 0.05 (Student’s *t*-test) were utilized to identify significantly differential metabolites, and Kyoto Encyclopedia of Genes and Genomes (KEGG) was used for pathway enrichment analysis.

### 2.9 Electron microscopy

A 1 mm^3^ volume of renal tissue was fixed overnight in 2.5% glutaraldehyde. After dehydration, embedding, curing and sectioning, ultrathin sections were stained with 2% uranyl acetate and lead citrate. Subsequently, cellular ultrastructural changes were observed under a transmission electron microscope (Hitachi HT7700, Tokyo, Japan).

### 2.10 Determination of MDA, 4-HNE and SOD in kidney tissues

The kidney tissues were homogenized in PBS and used for the determination of ferroptosis-related markers. The activity of superoxide dismutase (SOD) was measured by SOD activity assay kits (Elabscience Biotechnology Co., Ltd., Wuhan, China), and the levels of 4-hydroxynonenal (4-HNE) and malondialdehyde (MDA) were measured via MDA and 4-HNE assay kits purchased from Nanjing Jiancheng (Nanjing, China), respectively.

### 2.11 Quantitative reverse transcription-polymerase chain reaction (qRT-PCR)

0Kidney tissues were washed with PBS, and TRIzol reagent was used to extract the total RNA. The above RNA was reverse transcribed by Supermo III M-MLV reverse transcriptase to obtain the corresponding cDNA. PCR amplification was carried out using 2 × Power Taq PCR MasterMix and corresponding primers. The amount of PCR products was calculated using the fluorescence intensity of SYBR Green I, and the results were processed using the 2^−ΔΔCT^ method. These reagents were purchased from Beijing BioTeke Biotechnology Co., Ltd. (Beijing, China). Primer sequences were shown in Supplementary Table S1.

### 2.12 Western blotting analysis

Kidney tissues were added to RIPA buffer containing 1% PMSF, which were incubated at 4°C for 30 min. The proteins were obtained by centrifugation at low temperature. After separation, the protein samples were transferred to polyvinylidene difluoride membranes. Subsequently, the membranes were sealed with 5% skimmed milk and incubated with primary antibody overnight. After washing with TBST buffer solution, the membranes were incubated with secondary antibody. The primary antibodies were as follows: anti-GPX4 (Affinity Biosciences, Changzhou, China), anti-ACSL4 (Proteintech, Wuhan, China), anti-NCOA4 (Santa Cruz, Dallas, Texas, United States), anti-FTH1 (BOSTER Biological Technology Co., Ltd., Wuhan, China) and anti-β-actin (Proteintech, Wuhan, China).

### 2.13 Statistical analysis

GraphPad Prism 9.0 was used for statistical analysis. Data were presented as mean ± SD. One-way ANOVA was used to analyze the significance, followed by Dunnett’s test. *p* < 0.05 was considered statistical significant. Moreover, the potential relationships between gut microbiota, serum metabolites and host phenotypes were performed via Spearman correlation analysis.

## 3 Results

### 3.1 Chemical composition of kidney tea

Based peak intensity (BPI) chromatograms were shown in Supplementary Figure S1. Polyphenols are the main active ingredients of kidney tea (Chiang et al., 2023), and such compounds are considered to have antioxidant and nephroprotective effects (Jiang et al., 2012; Oršolić et al., 2021). Therefore, we identified the polyphenols in kidney tea. 12 polyphenols with relative percentages higher than 0.5% were identified by qualitative analysis (Table 1). Among them, the relative percentage of rosmarinic acid was the highest, accounting for 6.40%. The total content of 12 identified polyphenols in kidney tea reached 19.76%.

**TABLE 1:** Identification of polyphenols with relative percentages higher than 0.5% in kidney tea.

Compounds	Formula	PubChem ID	RT (min)	Ionization mode	m/z	Relative percentage (%)
Rosmarinic acid	C_18_H_16_O_8_	5281792	5.74	ESI−	359.0771	6.40
Butein	C_15_H_12_O_5_	5281222	5.82	ESI+	163.0386	3.75
Sagerinic acid	C_36_H_32_O_16_	23760102	5.75	ESI−	719.1622	1.61
Salvianolic acid B	C_36_H_30_O_16_	13991589	6.09	ESI−	717.1467	1.15
[2,6-dihydroxy-5-[3,4,5-trihydroxy-6-(hydroxymethyl)oxan-2-yl]oxycyclohex-3-en-1-yl] (E)-3-(3,4-dihydroxyphenyl)prop-2-enoate	C_21_H_26_O_12_	45359710	4.01	ESI−	179.0343	1.14
Odoratin	C_17_H_14_O_6_	13965473	10.51	ESI+	315.0859	1.11
(R)-3-(3,4-dihydroxyphenyl)lactate	C_9_H_10_O_5_	11600642	2.30	ESI−	197.0449	1.03
Eupatrin	C_18_H_16_O_7_	162464	10.48	ESI+	345.0966	0.93
Chicoric acid	C_22_H_18_O_12_	5281764	4.83	ESI−	473.0725	0.72
Caffeic acid	C_9_H_8_O_4_	689043	5.82	ESI+	181.0492	0.69
Lithospermic acid	C_27_H_22_O_12_	6441498	4.74	ESI−	537.1041	0.66
3,4-dihydroxybenzaldehyde	C_7_H_6_O_3_	8768	3.49	ESI−	137.0234	0.57

RT, retention time; m/z, parent ion mass-to-charge ratio; ESI+, positive ion mode; ESI−, negative ion mode.

### 3.2 Therapeutic effects of kidney tea on DN mice

**FIGURE 1:**
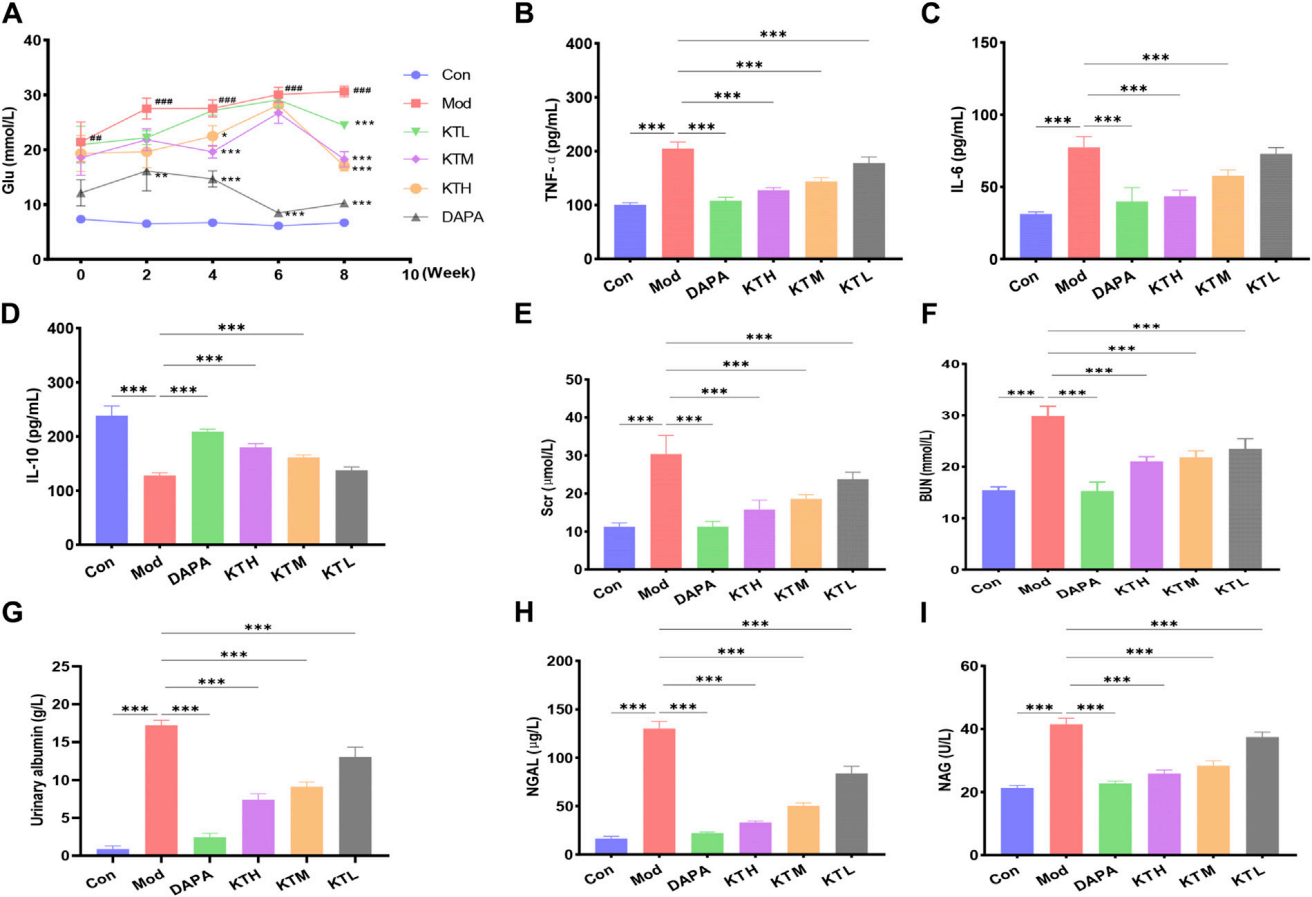
Effects of kidney tea on FBG, inflammatory cytokines and renal injury in DN mice. **(A)** Fasting blood glucose (FBG). **(B–F)** Serum TNF-α, IL-6, IL-10, Scr, and BUN levels. **(G–I)** Urine albumin, NGAL and NAG levels. ^#^ *p* < 0.05, ^##^ *p* < 0.01, ^###^ *p* < 0.001 compared with the Con group; *p < 0.05, **p < 0.01, ***p < 0.001 compared with the Mod group.

The animal experimental design was described in Supplementary Figure S2. First, we evaluated the therapeutic effects of kidney tea on DN mice via measuring FBG. Compared to normal mice, DN mice exhibited significant hyperglycemia, whereas kidney tea treatment reduced FBG levels to some extent (Figure 1A). Chronic inflammation is closely related to the pathogenesis of DN (Zhang et al., 2024). Therefore, the levels of the inflammatory factors IL-6, IL-10 and TNF-α were measured. The results found that TNF-α and IL-6 levels were significantly increased, while anti-inflammatory cytokine IL-10 levels were significantly decreased in DN mice (Figures 1B–D). High and medium doses of kidney tea could obviously reverse the levels of these cytokines. Moreover, renal injury-related markers including Scr, BUN and urinary albumin were measured. Ferroptosis has been reported to occur in renal tubular epithelial cells in diabetic nephropathy (Jin and Chen, 2022). Therefore, we also measured NGAL and NAG levels. The levels of Scr, BUN, urinary albumin, NGAL and NAG in the Mod group were significantly higher than those in the Con group, which could be significantly reduced by kidney tea treatment (Figures 1E–I).

**FIGURE 2:**
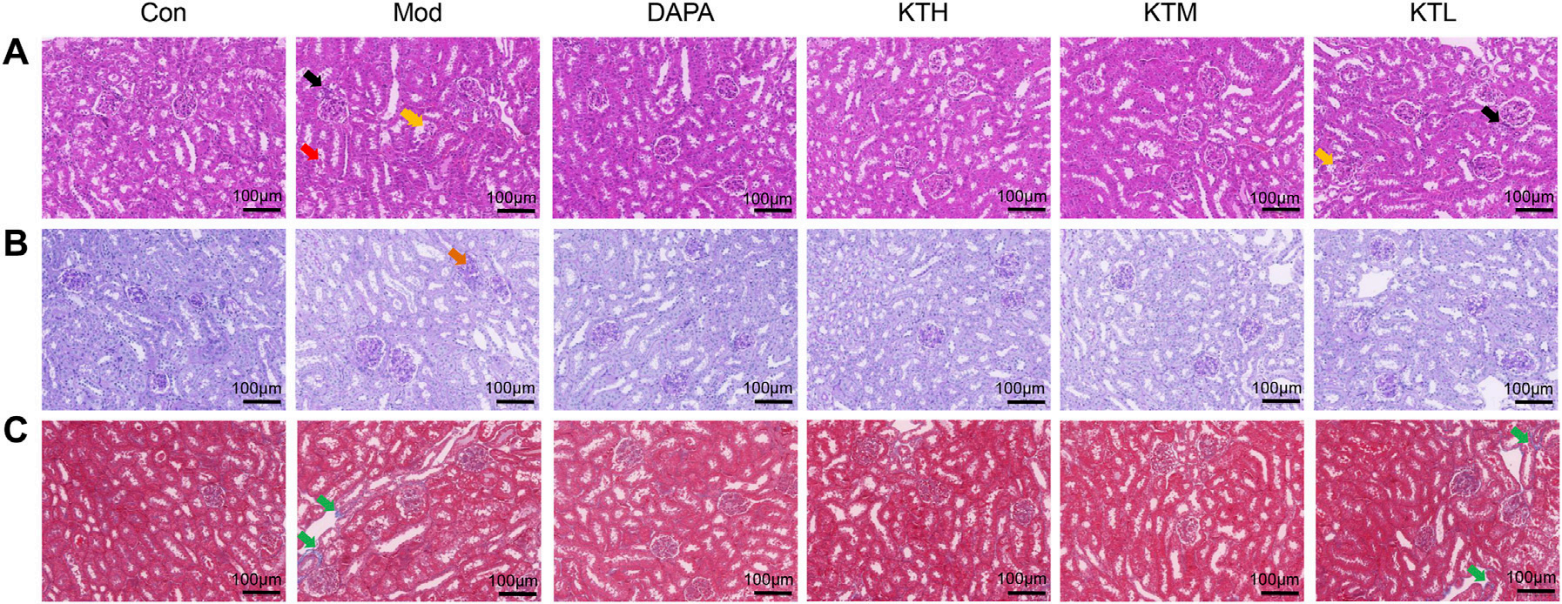
Effects of kidney tea on renal pathological changes in DN mice. **(A)** H&E, **(B)** PAS and **(C)** Masson staining of kidney samples (×200 magnification, scale bar: 100 μm). Black arrow: interstitial inflammatory infiltrate; yellow arrow: shrunken glomeruli; red arrow: massive degeneration of tubular epithelium; brown arrow: thickened glomerular basement membrane; green arrow: collagen fiber protein.

Next, the effects of kidney tea on renal morphological changes in DN mice were determined by H&E, PAS and Masson staining (Figures 2A–C). By H&E staining, shrunken glomeruli, interstitial inflammatory infiltrate, and massive degeneration of tubular epithelium were observed in DN mice (Figure 2A). PAS and Masson staining showed thickened glomerular basement membrane and increased collagen fiber proteins in DN mice, respectively (Figures 2B,C). Notably, these pathological changes were ameliorated after kidney tea treatment. Taken together, these results suggested that kidney tea improved renal function and injury in DN mice.

### 3.3 Kidney tea modulated the gut microbiota in DN mice

**FIGURE 3:**
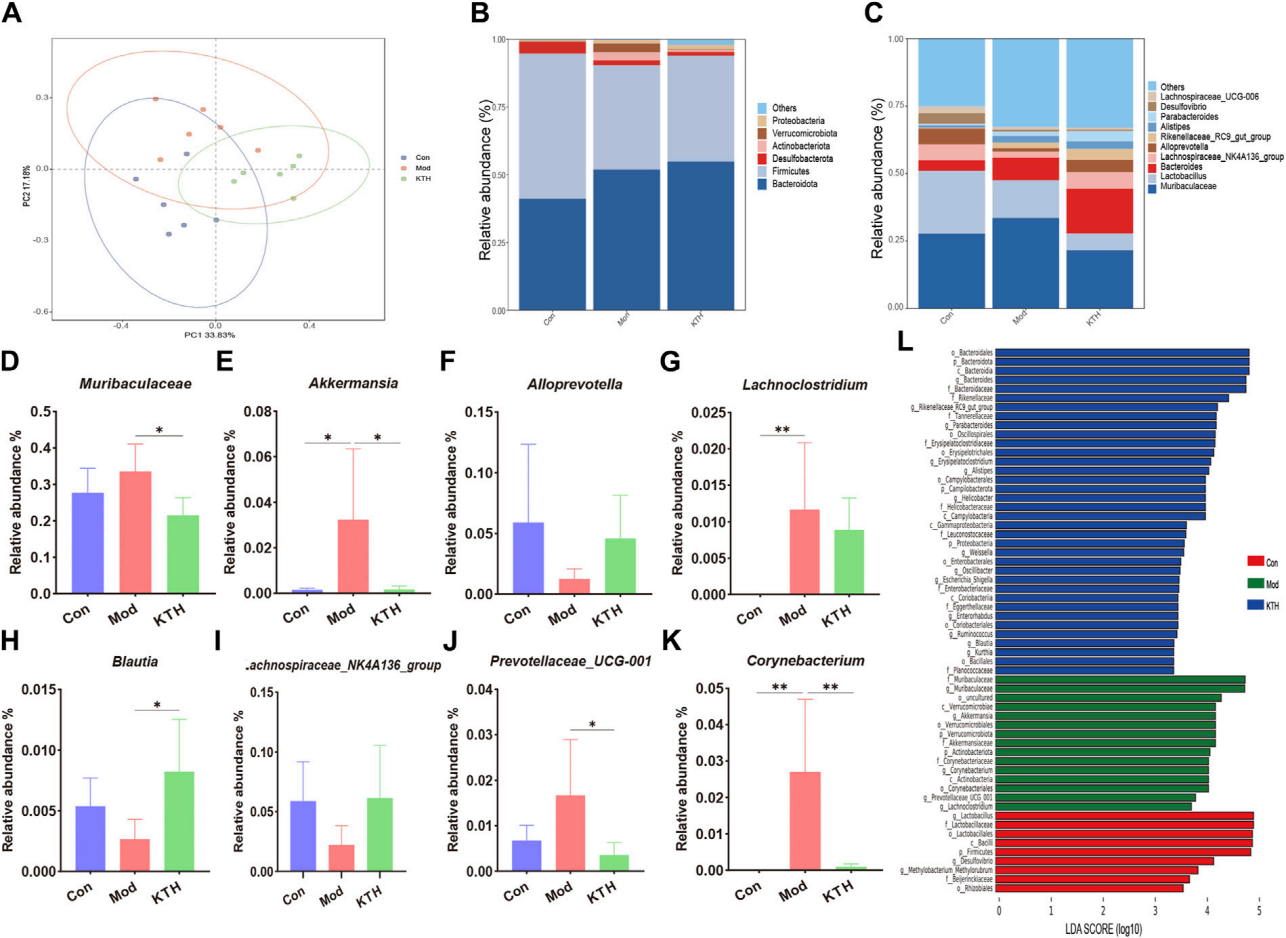
Kidney tea reshaped the gut microbiota in DN mice. **(A)** Principal coordinate analysis (PCoA) of the gut microbiota among all groups. **(B, C)** Relative abundance of the gut microbiota at the phylum and genus levels. **(D–K)** Relative abundance of eight key bacteria at the genus level. **(L)** Distribution histogram based on LDA score. *p < 0.05, **p < 0.01 compared with the Mod group.

Considering that DN mice treated with high-dose kidney tea showed the best renal protection, the KTH group was used for subsequent gut microbiota and metabolomics analysis. We first investigated the composition of the gut microbiota after kidney tea treatment by 16S rDNA gene sequencing. PCoA analysis found an obvious separation between the clusters of the Con, Mod and KTH groups (Figure 3A), indicating that kidney tea caused significant changes in the composition of the gut microbiota. At the phylum level, *Bacteroidota*, *Desulfobacterota*, *Firmicutes*, and *Actinobacteriota* were the dominant species among all of the groups (Figure 3B). Subsequently, we examined the structural alterations at the genus level (Figure 3C). The results revealed that the relative abundance of *Muribaculaceae*, *Lachnoclostridium*, *Akkermansia*, *Corynebacterium* and *Prevotellaceae_UCG-001* was increased, while the relative abundance of *Blautia*, *Alloprevotella* and *Lachnospiraceae_NK4A136_group* was decreased in DN mice compared to normal mice (Figures 3D–K). Significantly, kidney tea administration could reverse the dysbiosis of these bacterial taxa to some extent.

Furthermore, LEfSe analysis with an LDA score ≥ 3 was used to identify the characteristic bacterial taxa of each group (Figure 3L). Our results indicated that the significantly different microbiota in the Con, Mod and KTH groups were 9, 15 and 36, respectively. Of these, *f_Muribaculaceae*, *c_Verrucomicrobiae* and *g_Akkermansia* were the dominant microbiota in the Mod group. Importantly, high-dose kidney tea could enrich the relative abundance of *o_Bacteroidales*, *g_Bacteroides*, *f_Rikenellaceae*, *g_Rikenellaceae_RC9_gut_group*, and *f_Tannerellaceae*.

### 3.4 Kidney tea regulated serum metabolite profiles in DN mice

**FIGURE 4:**
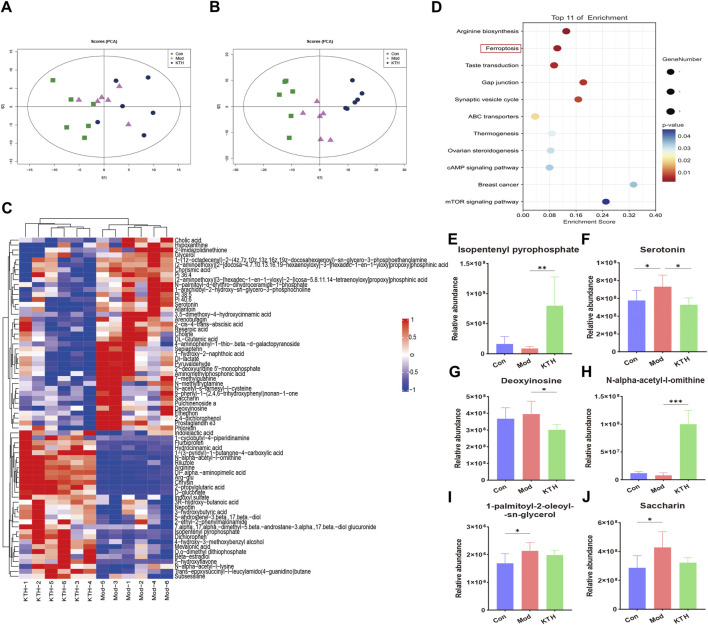
Kidney tea reversed the serum metabolomics profile in DN mice. **(A, B)** Principal component analysis (PCA) score plots of all samples under positive and negative ion modes. **(C)** Heat map of the top 50 serum differential metabolites. **(D)** Enrichment analysis of differential metabolites between the Mod group and the KTH group. **(E–J)** Relative abundance of six differential metabolites. *p < 0.05, **p < 0.01, ***p < 0.001 compared with the Mod group.

In addition, we used untargeted serum metabolomics to investigate the effects of kidney tea on host metabolites. The PCA plots showed the distinct separation of each group under positive and negative ion modes (Figures 4A, B). Meanwhile, VIP value > 1 and *p* < 0.05 were used as screening criteria for significantly differential metabolites. A total of 105 differential metabolites were identified in the Mod group compared with the Con group, indicating that the metabolite profile changed significantly during the development of DN. Compared with the Mod group, kidney tea intervention downregulated 41 metabolites and upregulated 31 metabolites. The heatmap showed the 50 most differentiated serum metabolites, implying that kidney tea exerted a profound effect on host metabolism (Figure 4C). Therefore, we further investigated the function of the differential metabolites between the two groups with pathway enrichment analysis. The results found that kidney tea mainly participated in the regulation of metabolic pathways such as ferroptosis, arginine biosynthesis, and mammalian target of rapamycin (mTOR) signaling pathway (Figure 4D). Among them, 14 differential metabolites were from the top 11 metabolic pathways (Supplementary Table S2). Notably, the relative abundance of serotonin, deoxyinosine, saccharin and 1-palmitoyl-2-oleoyl-sn-glycerol was increased, while the relative abundance of isopentenyl pyrophosphate and *N*-alpha-acetyl-l-ornithine was decreased in DN mice compared with normal mice (Figures 4E–J). Kidney tea intervention reversed the alterations of serum metabolites caused by DN.

### 3.5 Kidney tea alleviated ferroptosis in the kidneys of DN mice

**FIGURE 5:**
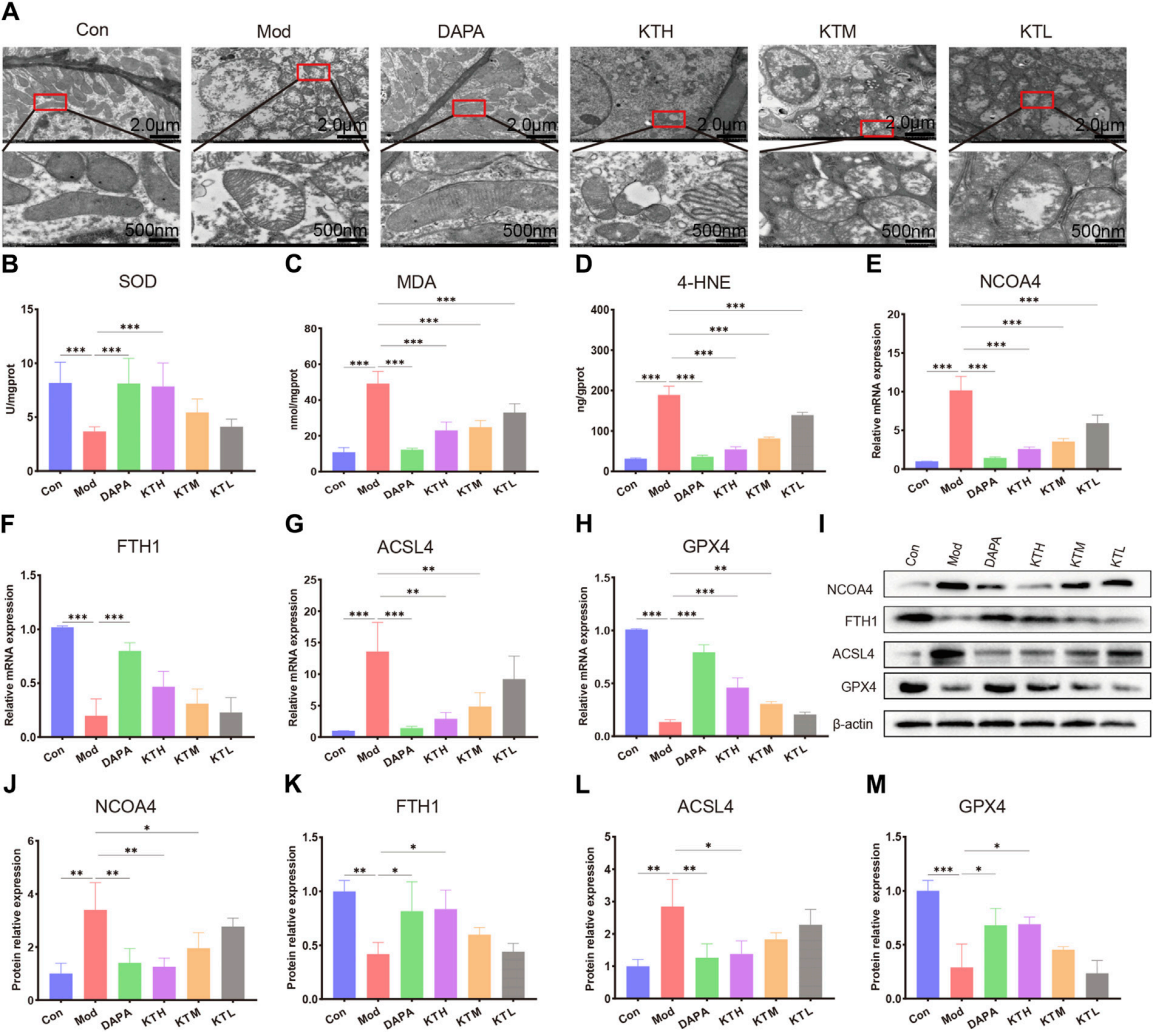
Kidney tea attenuated ferroptosis in the kidney. **(A)** Representative images of transmission electron microscopy of kidney samples (scale bar: 2 μm/500 nm). **(B)** SOD activity in kidney samples. **(C, D)** MDA and 4-HNE levels in kidney samples. **(E–H)** The mRNA expression of NCOA4, FTH1, ACSL4 and GPX4 in kidney samples. **(I–M)** The protein levels of NCOA4, FTH1, ACSL4 and GPX4 in kidney samples. *p < 0.05, **p < 0.01, ***p < 0.001 compared with the Mod group.

Based on the results of untargeted metabolomics, we further investigated the roles of kidney tea in the regulation of ferroptosis pathway. Electron microscope analysis revealed destruction of mitochondrial cristae in renal tissues of DN mice, while high and medium doses of kidney tea significantly attenuated mitochondrial damage (Figure 5A). SOD is a key intracellular antioxidant system, whereas MDA and 4-HNE are indicators of lipid peroxidation. Therefore, the relationship between ferroptosis and DN was further explored by measuring SOD, MDA and 4-HNE levels. The Mod group had lower activity of SOD as well as higher levels of MDA and 4-HNE than the Con group (Figures 5B–D). These findings suggested the presence of ferroptosis in the kidneys of DN mice. Of note, these indicators were ameliorated after kidney tea intervention. Next, we determined the expression of the ferroptosis-related proteins acyl-CoA synthetase long-chain 4 (ACSL4), glutathione peroxidase 4 (GPX4), ferritin heavy chain 1 (FTH1), and nuclear receptor coactivator 4 (NCOA4) in renal tissues. The mRNA expression of NCOA4 and ACSL4 was significantly increased in DN mice compared to normal mice, while kidney tea could decreased their expression (Figures 5E,G). The mRNA expression of FTH1 and GPX4 was significantly decreased in DN mice, which was reversed by kidney tea administration (Figures 5F,H). Western blot was further utilized to detect the expression of these proteins, and the results were consistent with qRT-PCR (Figures 5I–M). In conclusion, these data indicated that ferroptosis contributed to DN development, and that kidney tea played a therapeutic role in DN by improving ferroptosis in the kidneys.

### 3.6 Correlations between gut microbiota, metabolites and host phenotypes

**FIGURE 6:**
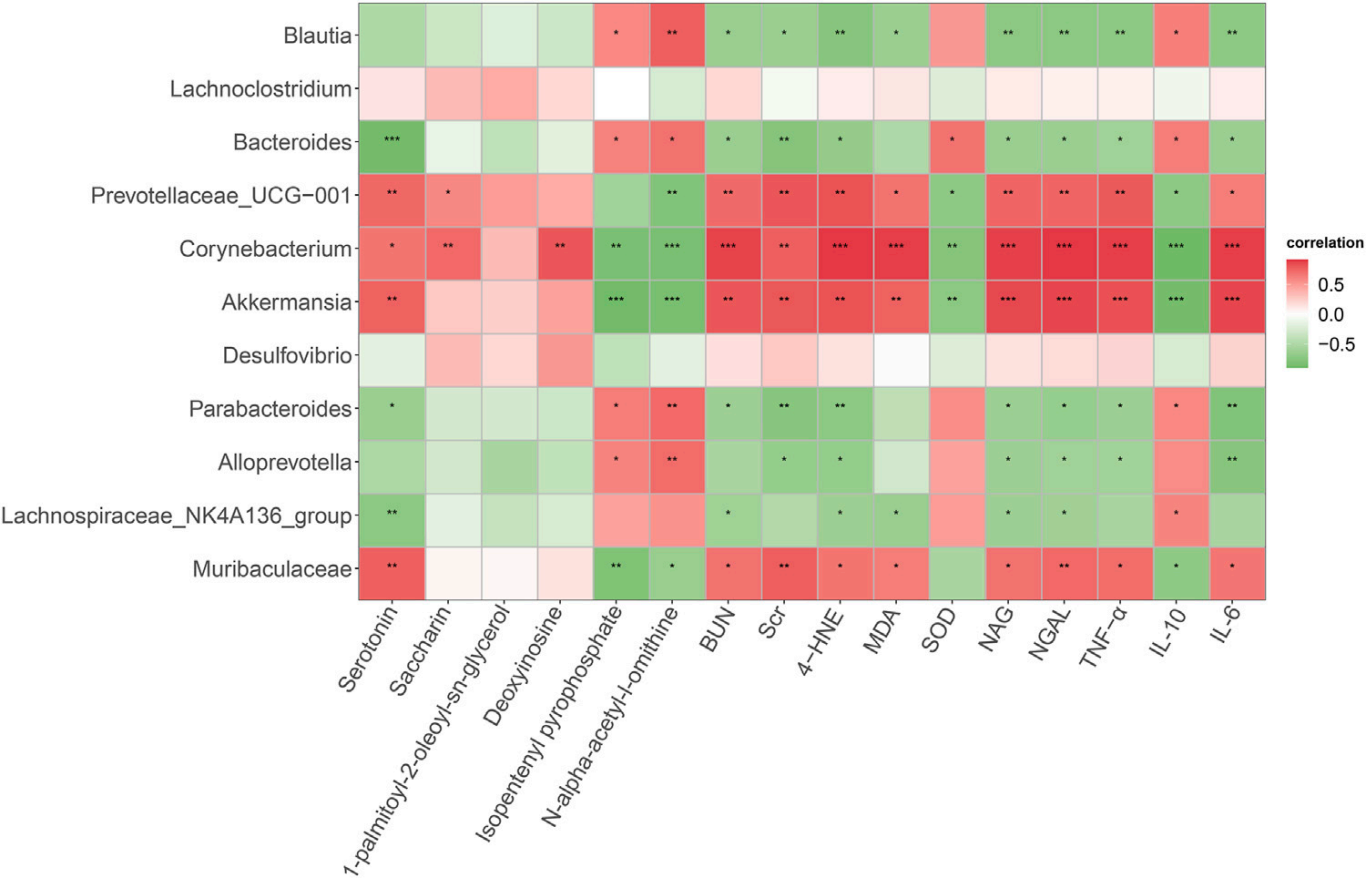
Spearman correlation analysis of the relationship between gut microbiota, serum metabolites, and host phenotypes. Red indicates a positive correlation and green indicates a negative correlation. *p < 0.05, **p < 0.01, ***p < 0.001.

Spearman correlation analysis was conducted to determine the potential relationships between differential bacterial genera, altered metabolites, and host phenotypes (Figure 6). Our results found that *Corynebacterium* and *Akkermansia* were positively correlated with serotonin but negatively correlated with isopentenyl pyrophosphate and *N*-alpha-acetyl-l-ornithine. Meanwhile, *Bacteroides* was significantly and negatively correlated with serotonin. In addition, *Prevotellaceae_UCG-001*, *Corynebacterium*, *Akkermansia*, and *Muribaculaceae* were positively correlated with renal injury indicators (Scr, BUN, NAG and NGAL), pro-inflammatory cytokines (IL-6 and TNF-α) and ferroptosis markers (MDA and 4-HNE), but negatively correlated with SOD and IL-10. On the contrary, *Blautia* and *Bacteroides* had an opposite relationships with these phenotypes. Altogether, these data supported that some key gut microbiota might be related to host metabolites and DN development.

## 4 Discussion

Diabetes is a chronic metabolic disease characterized by hyperglycemia. In recent years, the prevalence of diabetes has been increasing with the changes in diet and lifestyle (Mangione et al., 2022). At the same time, diabetes-associated complications have become a health issue of great concern to the global community. Among them, DN is one of the most important microvascular complications. Kidney tea is widely used for DN treatment, while studies on the mechanism of action are still lacking. The present study revealed that kidney tea significantly reduced the levels of FBG in DN mice, which was consistent with other studies (Lokman et al., 2019; Nguyen et al., 2019). Importantly, kidney tea could also reduce the levels of Scr, BUN, urinary albumin, NGAL and NAG, suggesting that this herb showed good nephroprotective effects. Increasing evidence suggests that the inflammatory response plays a vital role in the development of DN (Shahzad et al., 2022; Wang et al., 2023; Huang et al., 2023). WNT1-inducible signaling pathway protein 1 (WISP1) activated inflammatory responses in the kidney through the nuclear factor-kappa B (NF-κB) pathway, which was characterized by the increased expression of CCL2, TNF-α and IL-6 (Wang et al., 2022). Recent studies have found that oxidative stress and inflammation promote tubulointerstitial fibrosis, whereas activation of exchange protein activated by cAMP (Epac) can attenuate the progression of DN by modulating the C/EBP-β/SOCS3/STAT3 pathway (Yang et al., 2022). Our results showed that kidney tea intervention significantly reduced the levels of TNF-α and IL-6 and increased the levels of IL-10, suggesting that kidney tea attenuated inflammation in DN mice. Additionally, H&E, PAS and Masson staining confirmed that kidney tea ameliorated renal pathological damage. Collectively, these results suggest that kidney tea treatment can improve glucose metabolism and renal injury in DN mice.

Emerging evidence supports the involvement of the gut microbiota in the occurrence and progression of DN (Jiang et al., 2022; Zhang et al., 2023; Lu et al., 2023). Dysbiosis of gut microbiota resulted in increased acetate levels in DN rats, which promoted cholesterol homeostasis dysregulation and tubulointerstitial injury through activating G protein coupled receptor 43 (GPR43) (Hu et al., 2020). Furthermore, hyperactivation of complement C5 exacerbated renal dysfunction in db/db mice by promoting STAT3 expression and gut microbiota dysbiosis (Li et al., 2021). In the present research, kidney tea remodelled the gut microbiota in DN mice, manifested by an increase in the relative abundance of genera *Blautia*, *Alloprevotella* and *Lachnospiraceae_NK4A136_group*, as well as a decrease in the relative abundance of genera *Muribaculaceae*, *Lachnoclostridium*, *Akkermansia*, *Corynebacterium* and *Prevotellaceae_UCG-001*. *Blautia* are a group of anaerobic bacteria commonly considered to have probiotic properties. In DN mice with mild proteinuria, *Blautia* was negatively correlated with 24 h urinary protein levels (Li et al., 2020). Zhang et al. (2022) demonstrated that lycoperoside H could improve blood glucose and renal function in streptozotocin-induced DN rats by increasing the content of *Blautia* and *Ruminococcaceae*. Dysregulation of *Alloprevotella* is strongly associated with various pathological and physiological processes, such as atherosclerosis, ulcerative colitis and obesity (Kong et al., 2019; Liu et al., 2020; Wang et al., 2022). Compared with healthy individuals, both T2DM patients and DN patients have lower abundance of *Alloprevotella* and *Megasphaera* (Yang et al., 2022). Recent studies have found that resveratrol attenuates inflammation and kidney injury in db/db mice, and the mechanism is related to the elevated content of *Alloprevotella*, *Bacteroides* and *Parabacteroides* (Cai et al., 2020). Analogously, astaxanthin could protect against streptozotocin-induced DN by elevating the abundance of *Lachnospiraceae_NK4A136_group* (Ha et al., 2023). Consistent with these results, kidney tea might exert nephroprotective effects by enriching *Blautia*, *Alloprevotella* and *Lachnospiraceae_NK4A136_group*.

In addition to increasing the abundance of beneficial bacteria, kidney tea intervention inhibited the development of DN by decreasing the abundance of certain harmful bacteria. The diversity of the gut microbiota was lower in DN patients, and the relative abundance of *Lachnoclostridium* and *Bacteroides* was positively correlated with cholesterol levels (Chen et al., 2021). Moreover, *Morchella esculenta* polysaccharides might improve glycolipid metabolism and endotoxemia in T2DM mice by reducing the relative abundance of opportunistic bacteria such as *Corynebacterium* and *Actinobacteria* (Rehman et al., 2022). These data suggest that the reduced abundance of *Lachnoclostridium* and *Corynebacterium* may be the molecular mechanism by which kidney tea improves DN. Strikingly, it remains unknown whether *Akkermansia* is a friend or a foe in DN progression. *Akkermansia* was positively correlated with blood glucose, urinary albumin/creatinine ratio, and kidney/body weight ratio in DN mice, whereas *Abelmoschus Manihot* attenuated renal injury by reducing the abundance of *Akkermansia* (Shi et al., 2023). On the contrary, the content of *Akkermansia* was increased in ginsenoside compound K-treated db/db mice (Chen et al., 2022). Therefore, further studies are needed to elucidate the roles of *Akkermansia* in the pathogenesis of DN.

Serum metabolomics analysis revealed that kidney tea significantly reversed metabolite dysregulation. A total of 72 differential metabolites were identified between the Mod group and the KTH group. Of these, 41 metabolites were downregulated and 31 metabolites were upregulated after kidney tea supplementation. Previous studies have found that lower serum L-arginine levels and creatinine clearance, as well as higher postprandial glucose levels may be reliable predictors of microalbuminuria development in T2DM patients (Bariş et al., 2009). L-arginine and β-caryophyllene combination could attenuate renal fibrosis and inflammation in diabetic rats by inhibiting NF-κβ (Kumawat and Kaur, 2023). The higher initial levels of 3-hydroxybutyric acid indicated better control of HbA1c during drug treatment in newly diagnosed T2DM patients (Lee et al., 2023). Another study demonstrated that 3-hydroxybutyric acid could improve insulin resistance in diabetic mice by regulating hydroxycarboxylic acid receptor 2 (HCAR2) (Zhang et al., 2023). In addition, metabolomics analysis revealed that the levels of allantoin were higher in the kidneys and urine of DN rats compared to control rats (Liu et al., 2015). Similarly, choline content was significantly elevated in the plasma of diabetic patients and positively correlated with blood glucose levels (Li et al., 2023). In the present research, kidney tea intervention elevated the levels of arginine and 3-hydroxybutyric acid and decreased the levels of allantoin and choline, suggesting that kidney tea might prevent the development of DN by regulating these key metabolites.

Importantly, our results showed that kidney tea mainly participated in ferroptosis, arginine biosynthesis and mTOR signaling pathway. Ferroptosis, an iron-dependent form of regulated cell death, is considered to be closely associated with DN pathogenesis (Guo et al., 2023; Kim et al., 2023; Tsai et al., 2023). Attractively, emodin inhibited ferroptosis by promoting the expression of nuclear factor E2-related factor 2 (NRF2), thereby reducing reactive oxygen species (ROS) production and ameliorating renal injury in DN rats (Ji et al., 2023). Recent research has found that vitexin can improve ferroptosis-mediated DN by up-regulating solute carrier family 7 member 11 (SLC7A11) and GPX4 (Zhang et al., 2023). By electron microscope analysis, we confirmed that kidney tea attenuated mitochondrial damage in DN mice. Meanwhile, kidney tea significantly increased SOD activity and decreased MDA and 4-HNE levels, indicating that this herb attenuated lipid peroxidation in kidney tissues. ACSL4 is involved in the biosynthesis of polyunsaturated fatty acid (PUFA)-phospholipid in the cell membrane, which drives the operation of ferroptosis (Dai et al., 2023). Ferritin consists of two types of subunits FTH1 and ferritin light chain (FTL), and is primarily responsible for intracellular iron storage (Zhang et al., 2021). NCOA4 caused iron overload by mediating ferritin degradation, thereby promoting lipid peroxidation and ferroptosis (Jin et al., 2023). Conversely, GPX4 is a key anti-ferroptosis factor that exerts its antioxidant effect by converting lipid hydroperoxides to lipid alcohols (Bersuker et al., 2019). Kidney tea treatment increased the expression of FTH1 and GPX4 and decreased the expression of ACSL4 and NCOA4 in the kidneys of DN mice. Collectively, these findings suggest that kidney tea may protect against the progression of DN by inhibiting ferroptosis.

## 5 Conclusion

**FIGURE 7:**
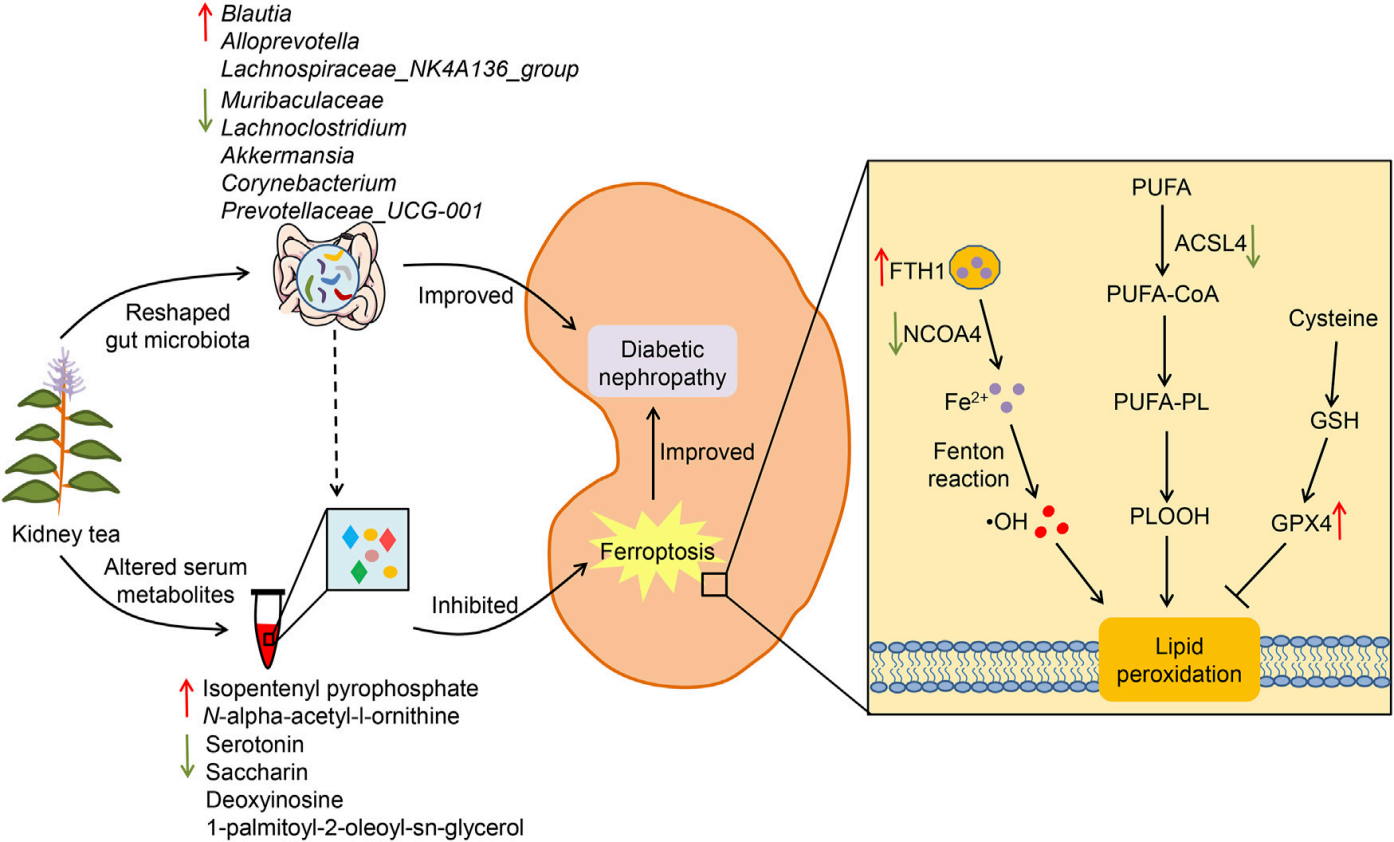
Potential mechanisms of kidney tea in improving DN.

In summary, our research demonstrates that kidney tea treatment attenuates glucose metabolism disorders, systemic inflammation, and renal injury in DN mice, and that the underlying mechanisms may be associated with the regulation of gut microbiota and metabolic profile (Figure 7). Meanwhile, kidney tea alleviates lipid peroxidation and ferroptosis in the kidneys by regulating the expression of NCOA4, FTH1, ACSL4 and GPX4. These findings provide a novel scientific rationale for the clinical application of kidney tea in DN. Future studies are needed to further clarify the active ingredients of kidney tea. Moreover, the specific relationship between gut microbiota and ferroptosis in the context of DN remains to be explored through fecal transplantation.

## Data Availability

The raw data has been deposited in valid repositories. The names of the repositories and accession numbers are as follows: https://www.ncbi.nlm.nih.gov/, PRJNA1121522; https://www.ebi.ac.uk/metabolights/, MTBLS10391.

## Abbreviations

DN, diabetic nephropathy; FBG, fasting blood glucose; SGLT2, sodium-glucose cotransporter 2; ARBs, angiotensin receptor blockers; ACEIs, angiotensin-converting enzyme inhibitors; GLUT4, glucose transporter 4; PI3K, phosphatidylinositol 3-kinase; AKT, protein kinase B; MS, mass spectrometry; UHPLC, ultrahigh-performance liquid chromatography; TNF-α, tumor necrosis factor-α; IL-6, interleukin-6; BUN, blood urea nitrogen; Scr, serum creatinine; NAG, N-acetyl-β-D-glucosaminidase; NGAL, neutrophil gelatinase-associated lipocalin; H&E, hematoxylin and eosin; PAS, periodic acid-Schiff; ASVs, amplicon sequence variants; PCoA, principal coordinates analysis; LEfSe, linear discriminant analysis effect size; OPLS-DA, orthogonal partial least squares discriminant analysis; PCA, principal component analysis; VIP, variable importance in the projection; KEGG, Kyoto Encyclopedia of Genes and Genomes; SOD, superoxide dismutase; 4-HNE, 4-hydroxynonenal; MDA, malondialdehyde; qRT-PCR, quantitative reverse transcription-polymerase chain reaction; BPI, based peak intensity; mTOR, mammalian target of rapamycin; ACSL4, acyl-CoA synthetase long-chain 4; GPX4, glutathione peroxidase 4; FTH1, ferritin heavy chain 1; NCOA4, nuclear receptor coactivator 4; WISP1, WNT1-inducible signaling pathway protein 1; NF-κB, nuclear factor-kappa B; Epac, exchange protein activated by cAMP; GPR43, G protein coupled receptor 43; HCAR2, hydroxycarboxylic acid receptor 2; SLC7A11, solute carrier family 7 member 11; PUFA, polyunsaturated fatty acid; FTL, ferritin light chain.
