# Supplementary material for: Kidney tea [Orthosiphon aristatus (Blume) Miq.] improves diabetic nephropathy via regulating gut microbiota and ferroptosis
Source: Front Pharmacol. 2024 Jun 19;15:1392123. doi: 10.3389/fphar.2024.1392123 (PMC11220284; doi:10.3389/fphar.2024.1392123)
Supplement: Supplementary file 1 [file DataSheet1.DOCX]

**Supplementary materials**

**Table S1** Primer sequences.

| Primers | Forward primer | Reverse primer |
| --- | --- | --- |
| ACSL4 | CCAGTGGCAGACTCGTAGC | AAGCCAGCAATAAAGTACACAGA |
| FTH1 | TTTGCAACTTCGTCGTTCCG | CAGGTTGATCTGGCGGTTGA |
| GPX4 | GTCCATTGGTCGGCTGCG | GGCTTAAGTAAGCGGCTCAGA |
| NCOA4 | AGATACATCTGCTCTGCGCC | GCCACTGGATGCTGACTTCT |
| GAPDH | AGGTCGGTGTGAACGGATTTG | TGTAGACCATGTAGTTGAGGTCA |

**Table S2** Differential metabolites in the top 11 metabolic pathways.

| Pathways | Differential metabolites |
| --- | --- |
| ABC transporters | Deoxyinosine, 1-palmitoyl-2-oleoyl-sn-glycerol, Arginine, Choline, DL-glutamic acid |
| Ferroptosis | Isopentenyl pyrophosphate, DL-glutamic acid, Mevalonic acid |
| Arginine biosynthesis | N-alpha-acetyl-l-ornithine, Arginine, DL-glutamic acid |
| Taste transduction | Saccharin, DL-glutamic acid, Serotonin |
| cAMP signaling pathway | 3-hydroxybutyric acid, Serotonin |
| Thermogenesis | 1-palmitoyl-2-oleoyl-sn-glycerol, Beta-estradiol |
| Ovarian steroidogenesis | 5-androstene-3beta,17beta-diol, Beta-estradiol |
| Gap junction | DL-glutamic acid, Serotonin |
| Synaptic vesicle cycle | DL-glutamic acid, Serotonin |
| mTOR signaling pathway | Arginine |
| Breast cancer | Beta-estradiol |


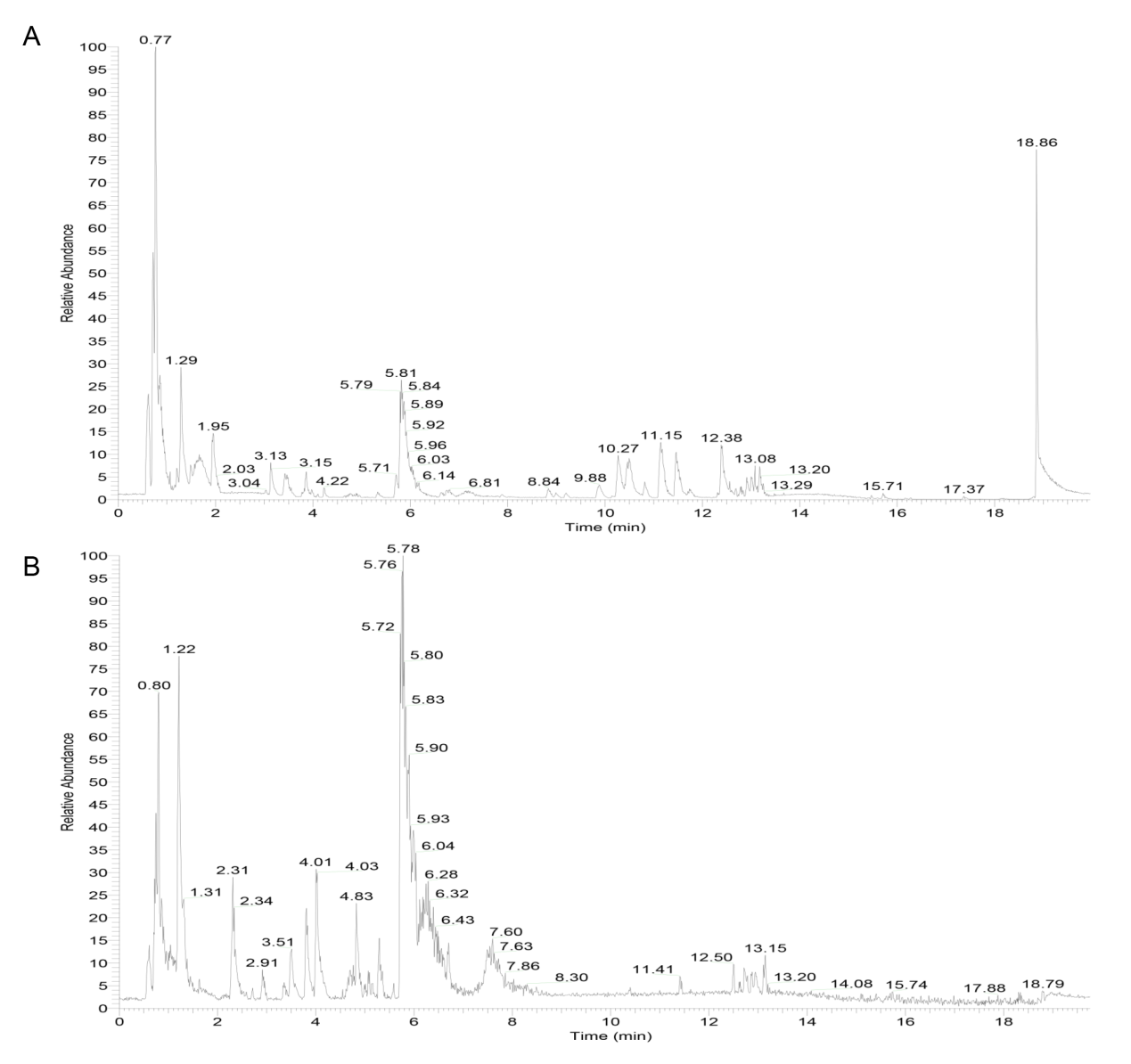


**Figure S1** UHPLC-MS analysis of the phytochemical composition of kidney tea. (A) Positive mode. (B) Negative mode.


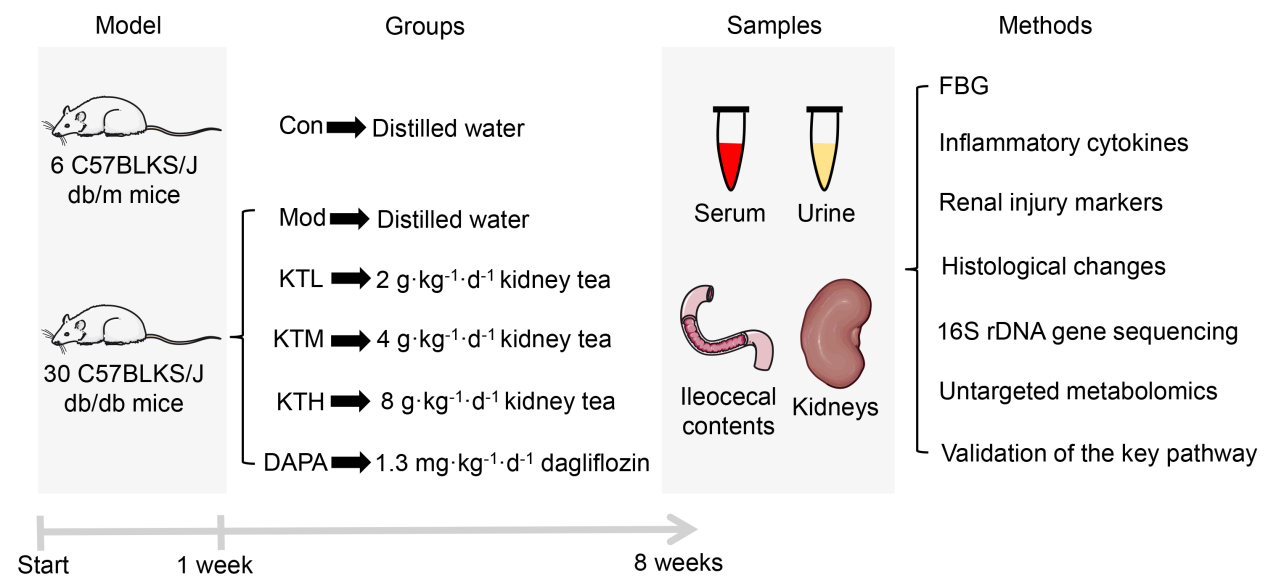


**Figure S2** The flow diagram of the experimental design.
